# Intensive Cognitive-Behavioral Therapy Telehealth for Pediatric Obsessive-Compulsive Disorder During the COVID-19 Pandemic: Comparison With a Matched Sample Treated in Person

**DOI:** 10.1016/j.jaacop.2023.09.007

**Published:** 2023-10-05

**Authors:** Martin E. Franklin, Jeffrey M. Engelmann, Nyssa Z. Bulkes, Gregor Horvath, Kelly Piacsek, Erik Osterlund, Jennifer Freeman, Rachel A. Schwartz, Michael B. Himle, Bradley C. Riemann

**Affiliations:** a Rogers Behavioral Health, Oconomowoc, Wisconsin; b Molson-Coors Beverage Company, Chicago, Illinois; c Rogers Behavioral Health, Oconomowoc, Wisconsin; d University of Michigan, Ann Arbor, Michigan; e Rogers Behavioral Health, Oconomowoc, Wisconsin; f University of Pennsylvania School of Medicine, Philadelphia, Pennsylvania; g Alpert Medical School at Brown University, Providence, Rhode Island; h University of Utah, Salt Lake City, Utah

**Keywords:** cognitive-behavioral therapy, intensive treatment models, multimodal treatment, pediatric OCD, telepsychiatry

## Abstract

**Objective:**

This naturalistic, nonblinded, nonrandomized study examined the efficacy of multimodal treatment including intensive cognitive-behavioral therapy (CBT) for pediatric obsessive-compulsive disorder (OCD) delivered via telehealth (TH) compared with a matched sample of youth treated in person (IP).

**Method:**

Patients included 1,286 youth ages 7 to 17 inclusive (643 TH, 643 IP) who received TH or IP in either partial hospitalization (n = 818) or intensive outpatient (n = 468) programs. Changes in patient-rated OCD symptoms and quality of life from pretreatment to posttreatment were examined.

**Results:**

TH patients were discharged with a statistically higher Children’s Yale-Brown Obsessive-Compulsive Scale Self-Report score than IP patients, although this group difference (1.4) was not clinically significant. Quality-of-life scores at discharge did not significantly differ between TH patients and IP patients. Treatment response was robust attesting to the broad applicability of the treatment model.

**Conclusion:**

Youth receiving CBT via TH responded both well and comparably to youth treated IP, offering a viable access path forward. These findings extend the reach of CBT for pediatric OCD. Concerted efforts must now be made to improve CBT availability for families for whom financial, insurance, geographical, and other barriers preclude access at present.

**Diversity & Inclusion Statement:**

We worked to ensure that the study questionnaires were prepared in an inclusive way. We worked to ensure sex and gender balance in the recruitment of human participants. We worked to ensure race, ethnic, and/or other types of diversity in the recruitment of human participants. We actively worked to promote sex and gender balance in our author group. While citing references scientifically relevant for this work, we also actively worked to promote sex and gender balance in our reference list. While citing references scientifically relevant for this work, we also actively worked to promote inclusion of historically underrepresented racial and/or ethnic groups in science in our reference list.

Telehealth (TH) involves the use of telecommunication technologies to provide health service availability across geographical distance and offers potential to expand access, increase quality, and reduce the spiraling costs of specialty health care services.^1^ Viewed as a means to bridge service gaps to rural communities across medical disciplines, interest in TH for psychology and psychiatry began when technologies to make it feasible were developed and improved.^2^ Accordingly, use of TH technologies in these clinical contexts became more widespread only in the last 2 decades.^3^ Multiple randomized trials attest to the efficacy of TH for a host of psychiatric and psychological conditions,^4^, ^5^, ^6^ providing further impetus to extend availability and test its limits with respect to diagnosis, patient characteristics, clinical contexts, and levels of care.

Obsessive-compulsive disorder (OCD) is a leading cause of disability in adults worldwide and is associated with family, social, and academic impairments in affected youth.^7^ Cognitive-behavioral therapy (CBT) involving exposure and response prevention has emerged as the treatment of choice around the world.^8^^,^^9^ CBT delivered via TH has proven efficacious for adults with OCD,^10^, ^11^, ^12^ and a burgeoning literature now supports its use for youth.^13^, ^14^, ^15^ Notably, most TH trials in OCD have examined weekly outpatient regimens, and thus it remains unclear whether TH is effective for individuals receiving higher levels of care, such as in an intensive outpatient program (IOP) or partial hospitalization program (PHP). These more intensive treatments are usually reserved for patients with greater severity, impairment, comorbidity, and a history of suboptimal response to weekly treatments. Moreover, unique challenges complicate TH use with youth requiring higher levels of care due to developmental factors, such as decreased attention span, capacity for self-direction, impulse control, and symptom insight. Such factors have been shown to decrease confidence of providers in their ability to mitigate therapy-interfering behaviors when delivering exposure plus response prevention therapy via TH.^16^ Studies examining TH outcomes in pediatric OCD also have considerable procedural variability, such as the use of patient- vs family-based approaches and methodological shortcomings (eg, use of no-treatment comparison conditions), which leave important questions unanswered, such as the relative effectiveness of TH vs care delivered in person (IP) for reducing OCD symptoms and improving quality of life for youth with OCD.

Use of TH for mental health services accelerated due to COVID-19, a highly contagious upper respiratory viral disease that led the World Health Organization to declare a global pandemic in March 2020.^17^^,^^18^ Nonessential workplaces, including mental health clinics, were shut down globally, forcing many health care providers to pivot to TH.^19^ With TH as the only option during the pandemic, the lack of data from large studies examining the effectiveness of TH for youth treated for OCD, especially those receiving a higher level of care, represented a critical gap in the extant literature.

The primary aim of this study was to compare treatment response for patients receiving TH with a matched sample receiving the same protocol delivered IP. The current study leverages a large sample of youth (N = 1,286) receiving IOP (n = 468) or PHP (n = 818) levels of care to examine predictors and moderators of treatment response (eg, demographic factors). Apart from the NORDLOTS trial (N = 269),^20^ no published trials examining any treatments for pediatric OCD have employed a sample size larger than 200, which underscores the potential for this study to advance the literature on this pressing topic.

Challenges associated with remote treatment have been described, including both general factors (eg, lack of therapist training in TH, concerns about establishing rapport, technological limitations, difficulty sharing therapeutic materials, patient distraction, Zoom fatigue) and factors that affect exposure therapy for OCD specifically (eg, conducting out-of-home exposures, managing patient distress/avoidance).^21^^,^^22^ Surveyed providers perceive TH as less feasible for more severe cases,^16^^,^^23^ but to date no systematic investigations have addressed these concerns. Given these challenges, we hypothesized that patients receiving TH would report less improvement in OCD symptoms and quality of life and show lower response and remission rates compared with patients receiving IP. We conducted a retrospective analysis of clinical data from a large, multistate integrated mental and behavioral health care system to test these hypotheses.

## Method

This naturalistic, nonblinded, nonrandomized study received exempt determination by the Institutional Review Board at Rogers Behavioral Health because it contained deidentified retrospective data analysis only and no prospective data collection.

### Participants and Procedure

Rogers Behavioral Health is a US-based multistate integrated mental and behavioral health care system that provides comprehensive multilevel care (intensive outpatient, partial hospitalization, and residential) for pediatric and adult patients with OCD. Retrospective, deidentified data were requested from an honest broker. The honest broker is an independent department at Rogers Behavioral Health that uses Institutional Review Board–approved procedures for providing deidentified data to researchers. Data are provided in a way that ensures that the research team cannot access codes linking deidentified data to the participants’ identifying information. Data were requested from the honest broker using the following patient inclusion criteria:

1. Treatment began between September 15, 2015 and September 15, 2022
2. Enrollment in the pediatric OCD program at the IOP or PHP level of care
3. Inclusive age range 7-17
4. Primary diagnosis of OCD
5. Admission score ≥16 on the self-report version of the Children’s Yale-Brown Obsessive-Compulsive Scale (CY-BOCS-SR)
6. Treatment delivered exclusively via TH or IP (ie, participants who transitioned from IP to TH at any time during treatment were excluded)
7. Completed CY-BOCS-SR at both admission and discharge.

This search yielded a sample of 1,649 patients, 643 receiving treatment delivered via TH and 1,006 receiving treatment delivered via IP. All patients in the TH group received treatment after June 1, 2020; patients who received IP care received treatment as early as September 28, 2015, and as late as March 2020. Because we required a discharge CY-BOCS-SR for analysis, dropout rates for the IP and TH groups were not known. Because medication was managed and optimized individually on an ongoing basis as a part of the multimodal treatment (for patients who received pharmacotherapy), the nuanced data necessary to examine medication effects were also not available.

The 643 patients treated via TH were matched to an equal-sized group of patients treated IP (from the identified eligible sample of 1,006 patients) based on age and level of care (PHP vs IOP). These factors were selected a priori based on our clinical experience that OCD symptoms tend to differ on both factors, with more severe CY-BOCS-SR scores at the more intensive PHP level of care and the tendency for CY-BOCS-SR scores to increase with age^24^; these same investigators also identified developmental differences in comorbidity patterns, associated impairment, insight, and type of symptoms experienced. We thus deemed it important to ensure that the groups were matched on age and level of care. Matching was conducted using the MatchIt package for R statistical software (R Foundation for Statistical Computing, Vienna, Austria).^25^ The algorithm identified 1:1 nearest neighbors based off propensity scores from a logistic regression model where treatment modality (IP, TH) was predicted using level of care (PHP, IOP) and age. Before matching, 683 of the 1,006 patients (67.9%) in the IP group were enrolled in PHP care and 409 of the 643 (63.6%) patients in the TH group were enrolled in PHP care (standardized mean difference = 0.09). Mean (SD) age was 13.7 (2.5) years for IP participants and 14.2 (2.3) years for TH participants, with a standardized mean difference between the 2 groups of 0.21 and a variance ratio of 0.87. Average distance metric from the logistic regression, which measures the degree of similarity between participants within each group based on both factors of interest (age and level of care), was 0.39 for the IP group and 0.40 for the TH group, with a standardized mean difference between the groups of 0.23 and a variance ratio of 0.92. After matching, there was an equal number of patients in PHP care in both the TH group and the IP group (409 of 643 for each group; 63.6%), and the mean (SD) age (14.2 [2.3] years) was also equal for the 2 groups. Average distance metric in both groups was 0.39 after matching. Thus, matching allowed us to retain common ratios of age and level of care across IP and TH groups.

### Assessment

Patients’ symptoms were assessed via telephone by admissions staff before admitting. Licensed psychiatrists and psychologists with expertise in OCD and other common psychiatric comorbidities (eg, depression, attention-deficit/hyperactivity disorder) reviewed phone screenings to determine eligibility and recommend level of care and to identify which treatment program was most appropriate. On admission, a child and adolescent psychiatrist completed a diagnostic interview with the patient to confirm any diagnoses using *DSM-5*.^26^

### Multimodal Treatment

Patients in IOP and PHP received multimodal treatment that involved individual CBT for OCD (with a primary emphasis on exposure plus response prevention), group CBT, family sessions, and medication management for patients receiving concomitant pharmacotherapy. For patients in PHP, treatment was delivered for 6 consecutive hours per day, 5 days per week. For patients in IOP, treatment was delivered for 3 consecutive hours per day, 5 days per week. Pharmacotherapy was managed by licensed child and adolescent psychiatrists or nurse practitioners. Psychotherapeutic interventions were administered by trained interventionists supervised by licensed clinicians with expertise in CBT. All interventionists were trained in delivering evidence-based therapy for OCD through participation in a didactic training course known as the CBT Academy. For each treatment arm, protocol adherence was promoted via use of treatment manuals, participation in regular clinical training opportunities, assessment and monitoring of therapist and treatment site effect sizes over time, and ongoing clinical supervision provided by experts in OCD phenomenology and the cognitive-behavioral interventions being delivered. Concerted efforts were made to deliver treatment comparably across TH and IP groups; however, some procedural adjustments were necessitated by the contextual differences in the 2 delivery modalities. For example, although patient-driven exposure and response prevention was emphasized in both modalities, therapists directly assisted with daily in-session exposure exercises in IP, whereas parents/caregivers in TH were encouraged, whenever possible, to provide support and assistance with exposure exercises conducted at home given that therapists in TH could participate remotely only.

### Measures

Patient-reported outcome measures were used to evaluate each patient at the start of treatment, biweekly throughout treatment, and again at discharge. The primary patient-reported outcome measures used to measure treatment outcomes were the CY-BOCS-SR and the Pediatric Quality of Life Enjoyment and Satisfaction Questionnaire (PQ-LES-Q).

#### Children’s Yale-Brown Obsessive-Compulsive Scale Self-Report

The CY-BOCS-SR^27^, ^28^, ^29^ is a self-report scale adapted from the assessor-rated CY-BOCS,^30^ which assesses OCD symptom severity in youth with 10 items, each on a 0–4 scale, addressing either obsessions or compulsions. Responses are summed for a total score ranging from 0 to 40, with higher scores indicating more severe OCD. Scores above 8 (mild) indicate the patient is likely to see reduced quality of life. Scores above 24 indicate moderate-to-severe OCD symptoms.^31^ At baseline, 44.0% (n = 566) of this sample met this severity cutoff, and 10.9% (n = 140) met this cutoff at discharge. The mean (SD) CY-BOCS-SR score at baseline was 23.9 (5.12) for the TH sample and 24.1 (5.20) for the IP sample. Internal consistency for the CY-BOCS-SR in this sample was acceptable (α = .78 at baseline and α = .92 at discharge). In a previous study examining reliable change on the Yale-Brown Obsessive-Compulsive Scale (Y-BOCS) and CY-BOCS,^32^ a difference on the instrument’s total score of 1.96 or greater constituted reliable change. A score of 16 on the instrument represents the lower end of moderate severity and accordingly has been used as the OCD symptom severity inclusion criterion for most of the seminal pediatric OCD treatment trials conducted over the past 20 years.^20^^,^^33^, ^34^, ^35^

#### Pediatric Quality of Life Enjoyment and Satisfaction Questionnaire

The PQ-LES-Q is a 15-item self-report questionnaire for youth ages 6 to 17 based on the original Quality of Life Enjoyment and Satisfaction Questionnaire (Q-LES-Q),^36^ assessing degree of enjoyment and satisfaction in daily functioning and life. Items are rated on a 5-point Likert scale with higher scores indicating better enjoyment and satisfaction. The first 14 items are summed to yield a total score that ranges from 14 to 70 and is expressed as a percentage of the items completed (0–100). Although a score between 70 and 100 is classically used as the normative cutoff for the Q-LES-Q,^37^ no similar cutoff was established for the PQ-LES-Q. Internal consistency for the PQ-LES-Q in this sample was good (α = .88 at baseline and α = .92 at discharge).

### Analytic Plan

All statistical analyses were carried out using R statistical software. The primary outcome measures were CY-BOCS-SR and PQ-LES-Q scores at admission and discharge and length of stay (measured in treatment days). Simple effects of treatment modality (IP vs TH) were assessed for each measure using Welch two-sample *t* tests. Bonferroni correction was used to control for multiple comparisons, such that the significance threshold for each test was set at *p* < .01. Effect sizes were computed using Cohen *d*.

To examine symptom improvement as a function of treatment modality, we conducted 2 linear regression models: one with discharge CY-BOCS-SR scores and another with PQ-LES-Q scores as the outcome variable. The predictors of interest were treatment modality (IP coded as 0, TH coded as 1) and level of care (IOP coded as 0, PHP coded as 1), and their interaction term to evaluate whether the effect of TH differed between IOP and PHP. The following covariates were included in the models: admission score, length of stay, age, sex assigned at birth (male, female), race (dummy-coded with White as the reference and a term for each of the following: Black, Native American/Alaska Native, Asian, Multiple, Native Hawaiian/Pacific Islander, or Other), ethnicity (not Hispanic = 0, Hispanic = 1), and comorbid diagnostic categories based on *DSM-5* criteria (1 indicated presence and 0 indicated absence of a diagnosis in each respective category).^26^ A diagnosis was included as a category in the model if it occurred in at least 20 participants. The following 7 diagnostic categories were included in the models: trichotillomania, excoriation disorder, depressive disorders, anxiety disorders, trauma-related disorders, feeding/eating disorders, and neurodevelopmental disorders. For these models, the significance criterion for each term was set at *p* < .05.

Secondary outcomes were assessed based on recent criteria for treatment response and remission in OCD.^38^ First, we created a binary flag for patients who experienced a symptom reduction of 35% or greater on the CY-BOCS-SR between admission and discharge (indicating treatment responder status). Second, we created a binary flag for patients whose discharge score on the CY-BOCS-SR was 12 or below (indicating remission status). We coded both as binary response variables and conducted separate binomial logistic regressions to probe if any of the predictors included in the linear regression models significantly suggested either the degree to which a patient’s symptoms would be reduced or whether they predicted a patient’s status as in remission. For these models, the significance criterion for each term was set at *p* < .05.

## Results

### Primary Outcome Measures

Descriptive statistics are presented in Table 1. Group means and standard deviations for each group and for the full sample are also presented in Table 1.

**Table 1:** Demographic Characteristics of In Person (IP) and Telehealth (TH) Groups

	IP (n = 643)				TH (n = 643)				All (N = 1,286)			
	n	Mean	(SD)	Range	n	Mean	(SD)	Range	n	Mean	(SD)	Range
Age, y	643	14.19	(2.31)	7-17	643	14.19	(2.31)	7-17	1,286	14.19	(2.31)	7-17
	**n**	**(%)**			**n**	**(%)**			**n**	**(%)**		
IOP	234	(36.39)			234	(36.39)			468	(36.39)		
Sex assigned at birth												
Male	288	(44.79)			212	(32.97)			500	(38.88)		
Female	355	(55.21)			429	(66.72)			784	(60.96)		
Not reported	0	(0.00)			2	(0.31)			2	(0.16)		
Race												
AI/AN	3	(0.47)			5	(0.78)			8	(0.62)		
Asian	24	(3.73)			16	(2.49)			40	(3.11)		
Black/AA	11	(1.71)			9	(1.40)			20	(1.56)		
Multiracial	16	(2.49)			13	(2.02)			29	(2.26)		
NH/PI	0	(0.00)			3	(0.47)			3	(0.23)		
Other	0	(0.00)			1	(0.16)			1	(0.08)		
White	516	(80.25)			445	(69.21)			961	(74.73)		
Not reported	73	(11.35)			151	(23.48)			224	(17.42)		
Ethnicity												
Hispanic	37	(5.75)			35	(5.44)			72	(5.60)		
Not Hispanic	513	(79.78)			510	(79.32)			1023	(79.55)		
Not reported	93	(14.46)			98	(15.24)			191	(14.85)		
Comorbid diagnostic categories												
Trichotillomania	14	(2.18)			19	(2.95)			33	(2.57)		
Excoriation disorder	9	(1.40)			15	(2.33)			24	(1.87)		
Depressive disorders	289	(44.95)			315	(48.99)			604	(46.97)		
Anxiety disorders	321	(49.92)			414	(64.39)			735	(57.15)		
Trauma-related	21	(3.27)			26	(4.04)			47	(3.65)		
Feeding/eating	50	(7.78)			55	(8.55)			105	(8.16)		
Neurodevelopmental	186	(28.93)			204	(31.73)			390	(30.33)		

	n	Mean	(SD)	Range	n	Mean	(SD)	Range	n	Mean	(SD)	Range
Diagnosis count	643	2.69	(1.24)	1-7	643	2.99	(1.32)	1-8	1,286	2.84	(1.29)	1-8
CY-BOCS-SR (A)	643	24.13	(5.19)	16-39	643	23.67	(5.12)	16-40	1,286	24.04	(5.15)	16-40
CY-BOCS-SR (D)	643	15.20	(7.43)	0-40	643	16.60	(6.67)	0-39	1,286	15.90	(7.41)	0-40
PQ-LES-Q (A)	591	57.11	(16.17)	1.79-98.21	590	57.56	(15.84)	14.29-100	1,181	57.34	(16.00)	1.79-100
PQ-LES-Q (D)	591	67.69	(17.45)	3.57-100	590	66.90	(16.04)	12.50-100	1,181	67.30	(16.76)	3.57-100
Length of stay	643	28.33	(13.46)	2-96	643	29.43	(13.85)	1-113	1,286	28.88	(13.66)	1-113

Note: For categorical variables, percentage of sample in group is presented instead of mean (SD). For continuous variables, n indicates number not missing; for categorical variables, n indicates number in that group. Diagnosis count is the total number of DSM diagnoses, including obsessive-compulsive disorder. Length of stay is measured in treatment days. (A) = admission; AI/AN = American Indian or Alaska Native; AA = African American; CY-BOCS-SR = Children’s Yale-Brown Obsessive-Compulsive Scale Self-Report; (D) = discharge; IOP = intensive outpatient program; NH/PI = Native Hawaiian or Pacific Islander; PQ-LES-Q = Pediatric Quality of Life Enjoyment and Satisfaction Questionnaire.

For CY-BOCS-SR score at admission, the IP and TH groups did not significantly differ (*t*_1283.8_ = 0.62, uncorrected *p* = .53, Cohen *d* = 0.03). At discharge, patients in IP had significantly lower CY-BOCS-SR scores than patients in TH (*t*_1269.5_ = −3.57, uncorrected *p* = .0004, Cohen *d* = 0.20). This corresponds to a small effect size using conventional guidelines of *d* = 0.2 for small, 0.5 for medium, and 0.8 for large. Differences in PQ-LES-Q between IP and TH did not significantly differ at admission (*t*_1178.6_ = −0.49, uncorrected *p* = .62, Cohen *d* = 0.03) or at discharge (*t*_1171.1_ = 0.81, uncorrected *p* = .42, Cohen *d* = 0.05).The 2 groups also did not significantly differ in length of stay (*t*_1282.9_ = −1.44, uncorrected *p* = .15, Cohen *d* = 0.08).

### Ordinary Least Squares Regression

Results are shown in Table 2. For the CY-BOCS-SR, participants in the TH group had higher discharge scores than participants in the IP group (β = 1.85, SE = .72, *t*_979_ = 2.56, uncorrected *p* = .01). There was no significant main effect or interaction involving level of care (|*t*|s < 1, *p*s > .40). Higher baseline scores (β = .46, SE = .04, *t*_979_ = 10.36, uncorrected *p* < .001), older age (β = .26, SE = .10, *t*_979_ = 2.70, uncorrected *p* = .007), and Asian race (β = 3.22, SE = 1.20, *t*_979_ = 2.69, uncorrected *p* = .007) were associated with higher discharge CY-BOCS-SR scores. Longer length of stay (β = −.07, SE = .02, *t*_979_ = −4.52, uncorrected *p* < .001) and comorbid trauma-related disorders (β = −2.26, SE = 1.13, *t*_979_ = −1.99, uncorrected *p* = .047) were associated with lower discharge CY-BOCS-SR scores.

**Table 2:** Regression Analysis Results

	Discharge scores: ordinary least squares				CY-BOCS-SR change: logistic regression			
	CY-BOCS-SR		PQ-LES-Q		35% Reduction		Score ≤12	
	β (SE)	*p*	β (SE)	*p*	β (SE)	*p*	β (SE)	*p*
Intercept	1.52 (1.82)	.40	40.07 (4.06)	< .001	−.23 (.56)	.68	1.77 (.59)	.003
TH	1.85 (.72)	.01	−1.00 (1.53)	.52	−.30 (.23)	.18	−.41 (.24)	.09
LOC	−.01 (.64)	.99	.71 (1.37)	.60	.28 (.20)	.16	.08 (.21)	.71
Admit score	.46 (.04)	< .001	.57 (.03)	< .001	.04 (.01)	.01	−.08 (.01)	< .001
LOS	−.07 (.02)	< .001	.14 (.03)	< .001	.02 (.01)	< .001	.02 (.01)	.003
Age	.26 (.10)	.01	−.51 (.20)	.01	−.10 (.03)	.001	−.05 (.03)	.08
Female	.27 (.48)	.58	.04 (1.02)	.97	.09 (.15)	.54	−.09 (.15)	.55
Black/AA	−1.22 (1.63)	.45	7.71 (3.50)	.03	.43 (.50)	.39	−.03 (.52)	.96
AI/AN	−.61 (2.60)	.82	−.38 (5.26)	.94	.93 (.85)	.28	.07 (.79)	.93
Asian	3.22 (1.20)	.01	−3.34 (2.61)	.20	−.90 (.41)	.03	−1.03 (.50)	.04
Multiple	1.02 (1.32)	.44	−1.30 (2.87)	.65	.004 (.40)	.99	−.42 (.45)	.35
NH/PI	.21 (3.96)	.96	−9.75 (9.80)	.32	.01 (1.24)	.99	−.04 (1.25)	.98
Other race	.48 (6.82)	.94	—	—	−12.67 (324.74)	.97	−13.07 (535.41)	.98
Hispanic	.07 (.93)	.94	.65 (1.99)	.74	−.04 (.28)	.90	−.07 (.31)	.82
Trichotillomania	2.26 (1.45)	.12	−5.37 (2.94)	.07	−.63 (.48)	.18	−.34 (.50)	.49
Excoriation	.12 (1.67)	.94	.45 (3.49)	.90	−.11 (.52)	.83	−.39 (.59)	.51
Depressive	.91 (.46)	.05	−2.02 (1.00)	.04	−.28 (.14)	.05	−.30 (.15)	.05
Anxiety	.45 (.46)	.33	−1.23 (.97)	.21	−.02 (.14)	.89	−.10 (.15)	.51
Trauma-related	−2.26 (1.13)	.05	1.11 (2.40)	.64	.44 (.35)	.22	.36 (.36)	.32
Feeding/eating	1.27 (.77)	.10	−4.29 (1.63)	.01	−.43 (.24)	.08	−.41 (.27)	.14
Neurodevelopmental	.69 (.49)	.17	−1.84 (1.05)	.08	−.09 (.15)	.57	−.20 (.16)	.22
TH × LOC	−.74 (.90)	.41	1.48 (1.91)	.44	−.04 (.28)	.89	.47 (.30)	.12

Note: The *p* values presented are uncorrected. AA = African American; AI = American Indian; admit score = score at admission; LOC = level of care; LOS = length of stay; NH = Native Hawaiian; PI = Pacific Islander; TH = telehealth; TH × LOC = telehealth × level of care interaction.

For the PQ-LES-Q, the effect of TH, the main effect of level of care, and the interaction between TH and level of care were not significant (|*t*|s < 1, *p*s > .43). Higher baseline scores (β = .57, SE = .03, *t*_896_ = 18.09, uncorrected *p* < .001), longer length of stay (β = .14, SE = .03, *t*_896_ = 4.13, *p* < .001), and Black/African American race (β = 7.71, SE = 3.50, *t*_896_ = 2.20, *p* = .03) were associated with higher PQ-LES-Q scores at discharge. Older age (β = −.51, SE = .20, *t*_896_ = −2.50, *p* = .01), comorbid depression (β = −2.02, SE = 1.00, *t*_896_ = −2.02, uncorrected *p* = .04), and comorbid eating/feeding disorders (β = −4.29, SE = 1.63, *t*_896_ = −2.64, uncorrected *p* = .009) were associated with lower PQ-LES-Q scores at discharge.

### Logistic Regression: Response and Remission

We defined treatment response as a reduction of at least 35% in CY-BOCS-SR score in line with recent criteria for treatment response in OCD.^38^ In the IP group, 330 of 643 patients (51.3%) met this criterion. In the TH group, 268 of 643 patients (41.7%) met this criterion. This difference was not statistically significant when taking covariates into account (β = −.30, SE = .23, *z* = −1.33, uncorrected *p* = .18, odds ratio = 0.74). Higher CY-BOCS-SR score at admission (β = .04, SE = .01, *z* = 2.80, uncorrected *p* = .005, odds ratio = 1.04) and longer length of stay (β = .02, SE = .01, *z* = 4.50, uncorrected *p* < .001, odds ratio = 1.02) were associated with higher likelihood of treatment response. Older age (β = −.10, SE = .03, *z* = −3.27, uncorrected *p* = .001, odds ratio = 0.91) and Asian race (β = −.90, SE = .41, *z* = −2.19, uncorrected *p* = .03, odds ratio = 0.41) were associated with lower likelihood of treatment response.

We defined remission as achieving a discharge score of 12 or lower on the CY-BOCS-SR, in line with criteria for treatment remission in OCD.^38^ In the IP group, 218 of 643 (33.9%) met this criterion. In the TH group, 187 of 643 (29.1%) met this criterion. The effect of TH was not significant (β = −.41, SE = .24, *z* = −1.71, uncorrected *p* = .09, odds ratio = 0.66). Longer length of stay was associated with a higher likelihood of remission (β = .02, SE = .01, *z* = 2.99, uncorrected *p* = .003, odds ratio = 1.02). Higher score at admission (β = −.08, SE = 0.01, *z* = −5.23, uncorrected *p* < .001, odds ratio = 0.92), Asian race (β = −1.03, SE = .50, *z* = −2.05, uncorrected *p* = .04, odds ratio = 0.36), and comorbid depressive disorders (β = −.30, SE = .14, *z* = −2.0, *p* = .048, odds ratio = 0.74) were associated with a lower likelihood of remission.

## Discussion

The current study contributes to a growing literature supporting the effectiveness of TH for OCD treatment.^13^^,^^14^ Broadly speaking, our findings encourage the use of TH as a treatment delivery modality for youth with OCD, which may expand access to care in settings in which IP care is not available or when care access is limited by barriers such as undue financial or time burden or public health crisis, as was the case during the COVID-19 pandemic. Barriers to empirically supported interventions for OCD have been noted as extending illness burden,^39^ and findings from this large naturalistic sample are convergent with outcomes reported in the broader literature. The specific role of TH in postpandemic OCD treatment remains to be seen, but the current study informs and supports ongoing efforts to determine how best to make use of the opportunities TH affords.^40^

It is noteworthy that the average discharge CY-BOCS-SR scores of patients receiving IP care were lower (1.4 points) than the scores of patients receiving TH care, which was consistent with our hypothesis that TH patients would show less improvement in OCD symptoms. Such a difference is unlikely to be observable in terms of OCD symptom severity and associated functional impairment, is within the one-tailed parameter of noninferiority (3.0 or less) established for a double-blind medication discontinuation phase for patients who had achieved wellness during acute treatment in a multisite randomized controlled trial,^41^ and does not exceed the parameters of reliable change on this instrument.^32^ Thus, this clinically trivial yet statistically significant difference does not obviate our broader conclusion about the utility of TH. However, it remains important to determine whether demographic and/or clinical variables moderate outcomes, which in turn can guide treatment decision making. Clinically, we suspect that very young children without sufficient parental assistance with treatment are more likely to struggle with TH given important developmental differences between this population and older children and adolescents, specifically with respect to the capacity to sustain attention and remain on task, self-motivation, and technical proficiency. Adapted TH protocols have been found to be effective for early-onset OCD,^13^ and such adaptations may be necessary for optimized outcomes with children younger than 10.

Regarding demographic predictors, Asian race was associated with poorer treatment outcomes, and African American race was associated with higher quality-of-life scores at discharge. The low number of minority participants and the large number of statistical tests conducted require cautious interpretation of these findings, however, and elucidate the need for future studies of treatment selection, delivery, modality, or levels of care among diverse demographic populations. The need to examine treatment differences by population was highlighted in a recent review, which noted that the dearth of comparable literature made it difficult to draw any evidence-based decisions on how to augment or individualize treatment based on patient characteristics.^42^ However, prior research has suggested that minority status, certain comorbidities (specifically motor and vocal tics), and peer and family can moderate OCD treatment; however, these findings were not borne out in meta-analytic findings, which instead identified some mixed support for moderation of patient age, comorbidities with anxiety disorders, individual vs group treatment, and total hours of treatment.^42^ The current study offers a relevant addition to the literature by testing treatment modality as another potential moderator of treatment outcomes and noting how it may interact with total hours of treatment, given the divergence between the IP and TH group only later in treatment. Ideally, future research will leverage similar large samples to further address gaps in understanding moderators of treatment efficacy, with a focus on those moderators noted by Kemp et al.^42^

Other predictors associated with outcome included length of stay, scores at admission, and certain comorbid diagnoses. Specifically, participants who stayed in treatment longer had better outcomes for both CY-BOCS-SR and PQ-LES-Q; participants who had more severe OCD symptoms at admission also had more severe symptoms at discharge and were less likely to achieve remission; and participants with higher quality-of-life scores at admission also had higher quality-of-life scores at discharge. Comorbid trauma-related disorders were associated with better outcomes on the CY-BOCS-SR; this finding was unexpected and should be interpreted with caution due to the near-threshold significance value (*p* = .047) and the large number of statistical tests for each model. Comorbid depressive and eating/feeding disorders were associated with poorer treatment outcomes. These findings should also be interpreted cautiously but are consistent with previous findings that certain comorbidities can weaken treatment outcomes for OCD^43^^,^^44^ and suggest that additional treatment might be needed for individuals with these comorbidities.^45^

A principal study limitation that must be acknowledged was the lack of random assignment to TH or IP conditions, which would have allowed for better control of confounding variables. Also, because we obtained data through an honest broker only for patients who completed treatment, we were unable to assess the number of participants excluded based on each criterion, and we were not able to assess dropout rates. Thus, the study findings reflect only the outcomes of patients who completed treatment and who were assessed at both admission and discharge. Indeed, the current study was a naturalistic one, borne of the necessity to pivot to TH due to the global pandemic; although important information can be gleaned, potential cohort or time effects were introduced as a result. Accordingly, randomized tests of TH vs IP in the treatment of pediatric OCD for patients treated at higher levels of care are needed to allow for more confident conclusions. Second, our sample was limited with respect to race and ethnic diversity, and thus questions about the possibility of differential response to IP vs TH in these groups remain unanswered. Subgroup analyses using advanced statistical methods are planned to explore such questions in detail. It is also the case that most participants were receiving concomitant pharmacotherapy during their multimodal treatment. The treatments themselves, including the use of active medication, were not blinded, and medication status was not controlled for in the analyses. Thus, the specific effects of the various treatment components cannot be isolated, and factors such as treatment expectation and response to pharmacotherapy could have contributed to the outcomes observed. A study examining the complex and potentially synergistic effects of medication-related variables is being undertaken by our research group. Further, although the CY-BOCS-SR has compared favorably to the assessor-rated version,^27^ the reliance on self-reported symptoms must be acknowledged as a potential source of imprecision.

Another limitation is that the psychotherapeutic interventions employed in this study were administered by interventionists who were extensively trained in delivering evidence-based therapy for OCD and who received ongoing monitoring and supervision by licensed clinicians to ensure protocol adherence. Such training has been shown to improve knowledge and adherence to evidence-based psychotherapy protocols,^46^^,^^47^ but risks limiting generalizability as studies have shown that exposure-based therapy techniques in particular are underused by community-based therapists,^48^^,^^49^ even among therapists who have received extensive training.^50^ A final limitation that warrants mention is that data regarding TH-related barriers and concerns (eg, the frequency of technological problems, therapist and patient perceptions of rapport) were not collected. Although recent studies have shown that most therapists report relatively high satisfaction with TH,^23^ some reservations remain, especially when delivering exposure plus response prevention for patients with more severe symptoms.^16^ Systematic investigations of challenges associated with TH delivery could inform protocol enhancements, allay concerns, and increase confidence in the adoption of TH to treat youth with OCD, including youth treated at higher levels of care.

These caveats notwithstanding, examining treatment outcome in the current cohort did afford opportunities to extend the literature. Youth receiving multimodal treatment at a higher level of care responded well and comparably when treatment was delivered via TH compared with IP, which offers a path forward for families who cannot readily access treatment due to commonly reported barriers such as lack of access to treatment providers, geographic location, or other personal or societal circumstances. Secondary analyses are planned to examine treatment response by modality in ethnic and racial minorities, effects of medication status on treatment outcome, and capacity for emerging statistical methodologies to maximize predictive power and hence identify subgroups of patients more or less likely to respond to treatment in general or to either treatment delivery modality specifically. Collectively, such findings will be useful in guiding treatment decision making and in strengthening the case for improving access to care, however it may be attained. OCD remains a pernicious illness with powerful negative effects on youth functioning that, without intervention, appear to extend into adulthood. Fortunately, viable options designed to extend treatment availability have been developed, tested, and refined. It is now incumbent on those of us in the field to address access barriers to these empirically supported interventions and hence alleviate associated suffering going forward.
